# Micronutrient Intake in a Cohort of Italian Adults with Type 1 Diabetes: Adherence to Dietary Recommendations

**DOI:** 10.1155/2017/2682319

**Published:** 2017-10-04

**Authors:** Marisa Giorgini, Marilena Vitale, Lutgarda Bozzetto, Ornella Ciano, Angela Giacco, Anna Rivieccio, Ilaria Calabrese, Gabriele Riccardi, Angela A. Rivellese, Giovanni Annuzzi

**Affiliations:** Department of Clinical Medicine and Surgery, Federico II University, Naples, Italy

## Abstract

Micronutrients are of fundamental importance in maintaining health status. However, data on their dietary intake are few particularly in persons with diabetes. The aim of this study was to evaluate in adults with type 1 diabetes (T1DM) attending a tertiary-level diabetes center in Southern Italy the intake of micronutrients (both vitamins and minerals) and the adherence to recommendations. Seven-day food records of 60 T1DM patients were analyzed. Micronutrient intake was evaluated based on the Italian food composition tables and expressed as amount per 1000 kcal of energy intake to adjust for possible underreporting. Adherence to recommendations for vitamins A, B_6_, B_12_, and C and niacin was acceptable in both sexes (ranging from 77% to 100%). Half of the patients did not adhere to folate recommendation, even less to vitamin E, and no patient reached the recommended intake for vitamin D. As for minerals, adherence was low for potassium and selenium (0–23%); intermediate for zinc, copper, and magnesium; low and intermediate for calcium in men and women, respectively; and low for iron in women. In conclusion, the diet followed by T1DM patients may not have a sufficient content of different micronutrients. Therefore, an adequate intake of low-fat dairy products, fish, legumes, and vegetables should be encouraged as components of a healthier dietary pattern.

## 1. Introduction

Besides macronutrients, micronutrients, in particular vitamins and minerals, are of fundamental importance in maintaining health status and in preventing and treating different diseases [1, 2]. Accordingly, precise dietary recommendations for each vitamin and mineral are issued for the general population. However, this may be even more important in people with diabetes, as different vitamins and minerals play a relevant role in the regulation of glucose metabolism at different levels (insulin action, oxidative stress, and inflammation) and in the prevention of diabetes complications [3]. In spite of the possible pathophysiological relevance of micronutrient levels, data on their dietary intake are few in the general population and really scant in persons with diabetes, especially those with type 1 diabetes [4–6].

In patients with type 1 diabetes, insulin therapy is essential for survival. At the same time, adequate nutrition therapy represents a cornerstone for reaching optimal glucose control and for preventing chronic complications. However, in these patients, diet therapy is essentially focused on the amount and quality of carbohydrate in order to regulate the doses of insulin to be injected [7, 8]. This focus may lead, both in caregivers and in patients, to less attention towards other macronutrients [9] and micronutrients. To this respect, while some data are present for diet composition in terms of macronutrients [6, 10–12], very little has been reported about micronutrients. In particular, one study refers to children with type 1 diabetes [5] and another to a Finnish adult population with type 1 diabetes [6].

Therefore, the aim of our study was to evaluate in adult patients with type 1 diabetes attending a tertiary-level diabetes center in Southern Italy the dietary intakes of micronutrients (both vitamins and minerals) and the adherence to the recommended dietary intakes.

## 2. Materials and Methods

All patients with type 1 diabetes attending the outpatient clinic of the Department of Clinical Medicine and Surgery of Federico II University, a tertiary-level diabetes care, from at least six months and consecutively visited within a period of six months were asked to fill in a seven-day food record, provided that they met the inclusion/exclusion criteria. The exclusion criteria were pregnancy, celiac disease, kidney failure (serum creatinine > 1.5 mg/dL), and other acute or chronic diseases apart from diabetes.

Detailed instructions on how to fill in the food records were given by a dietician. In particular, patients were asked to record for seven consecutive days the following:

- The amount of any food or drink consumed using a food scale or household measures
- The time of meals (breakfast, morning snack, lunch, afternoon snack, dinner, and evening snack)
- The type and, if possible, the brand of the product
- The cooking method

When the food records were given back by the patients, the dietician checked the records together with the patients in order to improve the completeness of data. Forms for food recording were given to 140 patients, but only 64 patients (46%) filled in and returned the food records. Of these, four were excluded for incompleteness, and therefore, the food records of 60 patients were analyzed.

The intake of water was not reported on the records by the patients. Since water intake is important especially in relation to calcium and magnesium intakes, we included in the calculations a fixed daily intake of 1000 mL of water for each patient, containing an estimated medium amount of calcium (150 mg) and magnesium (13 mg).

Energy intake and macro- and micronutrient contents were calculated on the basis of the Italian food composition tables of the Center of Research for Food and Nutrition (CREA) [13] utilizing the MetaDieta software (Meteda s.r.l., Ascoli Piceno, Italy).

The macronutrient composition, expressed as percentage of the total caloric intake, was compared with the dietary recommendations for diabetes given by the Italian Diabetes Standards of Care [14] that were mainly based on the recommendations of the Diabetes and Nutrition Study Group of the EASD [15].

Taking into account the possible underreporting, the intakes of vitamins and minerals are expressed as amount per 1000 kcal of total energy intake. Intakes were compared to the recommended levels expressed per 1000 kcal assuming an average daily intake of 1800 kcal for women and 2000 kcal for men. The percentage of the patients adhering to the recommendations given by the Italian Society of Nutrition [16] is reported.

Anthropometric measures and blood samples for determination of HbA1c and serum lipid concentrations were taken during the outpatient visit. Body weight measurement was performed using a scale provided with a weighing bar, with a precision of 0.1 kg, according to the standardized methods. The height was detected with a fixed stadiometer, and the measurement was performed with patients barefoot with the shoulders at the stadiometer. The body mass index was calculated by the ratio between the weight (kg) and the height squared (m^2^). The waist circumference was measured using a nonelastic meter between the inferior rib margin and the anterosuperior iliac spine after a deep expiration avoiding skin compression.

Glycated hemoglobin was measured by high-performance liquid chromatography (HPLC). Serum triglyceride and cholesterol concentrations were assayed by enzymatic colorimetric methods. LDL cholesterol concentration was calculated using Friedwald's formula.

## 3. Statistical Analysis

Data are expressed as mean ± SD. Differences between women and men were evaluated by *t*-test for independent samples. A *p* < 0.05 was considered statistically significant. The statistical analysis was performed according to standard methods using the Statistical Package for Social Sciences software, version 20 (SPSS/PC; SPSS, Chicago, IL, USA).

## 4. Results

The main characteristics of the participants (*n* = 60), divided by sex, are reported in Table 1. As expected, women had a lower waist circumference and higher HDL cholesterol levels. More than half of the participants (55%) were on multiple daily insulin injections (MDI) and the remaining ones (45%) on continuous subcutaneous insulin infusion (CSII). No differences for all the parameters considered were detected between MDI and CSII, and therefore, the two groups were combined for analysis. In Table 2, energy intake and diet composition are reported separately for men and women. The energy intake was quite low for both men and women. The intake of macronutrients was, on average, within the recommended dietary intake levels except for fiber consumption that was substantially lower than the recommended level. Sodium intake was lower than recommended, but it must be considered that only NaCl naturally present in foods was evaluated and not the amount added to foods.

The intakes of vitamins and minerals for men and women are shown in Table 3. In this table, also, the percentages of the patients adhering to the recommended dietary intakes are reported. The adherence is acceptable for both men and women for vitamin A, vitamin B_6_, vitamin B_12_, niacin, and vitamin C (ranging from 77% to 100%). However, half of the patients did not meet the recommended intake for folate, and the adherence was still lower for vitamin E (20% for men and 43% for women); furthermore, no patient reached the recommended intake for vitamin D. As for minerals, the adherence was extremely low for potassium (7% for men and 0% for women) and selenium (23% for both men and women) and intermediate for zinc, copper, and magnesium. The adherence for calcium and iron differed between men and women being for calcium low in men (50%) and intermediate in women (73%) and for iron high in men (97%) and low in women (57%).

## 5. Discussion

This study shows that the diet composition of our relatively small cohort of patients with type 1 diabetes was on average within the recommended dietary intakes for what concerns macronutrients. Fiber intake was, instead, very low confirming results obtained in patients with type 2 diabetes from the same geographical area [17, 18].

The main focus of our study was on micronutrient intake. To this respect, the most relevant results are that, even correcting for possible underreporting—a real problem in the evaluation of dietary habits whatever the method used—the adherence was unsatisfactory for some micronutrients.

In particular, as for vitamins, no patient adhered to the recommended intake for vitamin D. The adherence was poor for vitamin E and folate and, at least in women, for riboflavin. For what concerns minerals, the adherence was very poor for potassium, poor for selenium, and unsatisfactory for calcium (at least in men) and iron in women. For the other micronutrients, the adherence was in general not fully adequate but certainly more satisfactory.

Certainly, the most impressing results regard vitamin D as no patient adhered to the recommendations. This is in line with the data reported in the general population from different countries, for example, Italy, Finland, and USA [4, 19, 20], and in Finnish patients with type 1 diabetes [6]. To investigate about the reasons of this very low intake, we looked at the frequency of consumption of foods that represent the main source of vitamin D, that is, fish and dairy products. In our cohort, the consumption of fish (on average, one portion per week) was very low and that of dairy products (less than 2 servings per day) was lower than recommended (3 servings per day) by the USDA Dietary Guidelines for Americans [20].

The adherence to recommendations for potassium was very poor. This result may be related to the low consumption of vegetables and fruit in our population. For what concerns the other micronutrients for which we found poor or, anyhow, unsatisfactory adherence, intakes were lower compared to the ones reported in the only other study performed in adults with type 1 diabetes [6], although the results were quite in line with the results in the general population wherein unsatisfactory intakes for different vitamins (vitamin D, vitamin A, vitamin E, folate, and riboflavin) and minerals (calcium, magnesium, selenium, and iron) have been generally found [4]. The low intake of the above micronutrients in our population was linked, as for foods, especially to low consumption of fish and dairy products and also of legumes and vegetables as indicated by the low intake of fiber.

The poor adherence for some micronutrients in our population of patients with type 1 diabetes was rather unexpected. These patients receive precise dietary recommendations that should translate into a generally healthy diet. However, it is common that diabetes professionals and, even more, patients with diabetes focus their attention on the amount of carbohydrates on which they balance insulin therapy paying less attention to the overall diet, in particular to foods representing the main source of micronutrients. Furthermore, in order to avoid variations in postprandial blood glucose response, patients prefer to utilize a restricted selection of foods. This may contribute to making the diet not adequate in terms of micronutrient content.

Whatever the reason, the unsatisfactory intake of many important micronutrients in this particular category of patients could lead to a worsening of glucose control, a major susceptibility to micro- and macrovascular complications, and a higher risk of osteoporosis. This means that adequate strategies aiming at improving also micronutrient intake should be implemented in the general population and, particularly, in persons with type 1 diabetes. To this regard, it has been estimated that in the USA, the consumption of the daily recommended intake of dairy products would eliminate inadequacy of calcium intake across all age and gender categories and only partially improve vitamin A and magnesium intake. However, an adequate intake of dairy products would not reduce the poor adherence to vitamin D and potassium recommended intake, for which other strategies are needed such as sun exposure (if possible and with moderation) and increases in other foods rich in vitamin D (in particular fish) and potassium (legumes, vegetables, and fruit) [21].

Our study has some strengths, particularly in relation to the use of the 7-day food records that represent the gold standard for evaluating dietary habits at individual level. The limitation of the study is the possible underreporting that is anyway common to all types of food recording methods. In addition, the food record was filled in by the participants on only one occasion, and therefore, this did not allow to take into account the seasonal variability. Lastly, the results observed in this relatively small cohort should be confirmed in larger samples of patients with type 1 diabetes. To this respect, obtaining data on dietary habits in this category of patients may be challenging, as shown by the low percentage of people who filled in the records in the present study, in line with a similar participation rate in a previous study [6]. It can be speculated that patients who did not agree to participate had on the average a lower consideration for diet and therefore were less likely to be compliant to dietary recommendations. Consequently, if anything, the low participation rate may have hindered an even lower adherence to recommendations than that observed in our study.

In conclusion, our study underlines that the diet followed by patients with type 1 diabetes may not be adequate concerning micronutrient intake and that strategies aiming at solving this problem should be implemented. To this aim, an adequate intake of dairy products—in particular low-fat products in order to avoid an increment in saturated fat—fish, legumes, and vegetables should be encouraged as components of a healthier dietary pattern.

## Figures and Tables

**Table 1:** The main characteristics of the patients with type 1 diabetes participating in the study.

	All (*N* = 60)	Men (*N* = 30)	Women (*N* = 30)
Age (years)	35.8 ± 11.3	35.5 ± 11.1	36.0 ± 11.8
Diabetes duration (years)	17.3 ± 10.7	16.4 ± 10.5	18.4 ± 11.1
HbA1c (%)	7.6 ± 0.8	7.6 ± 0.9	7.7 ± 0.7
HbA1c (mmol/mol)	59.9 ± 9.3	59.5 ± 10.8	60.4 ± 7.8
Body mass index (kg/m^2^)	25.5 ± 3.7	25.7 ± 3.4	25.2 ± 4.0
Waist circumference (cm)	86.3 ± 10.9	90.1 ± 10.4	81.9 ± 9.9^∗^
HDL cholesterol (mg/dL)	62.7 ± 14.1	57.4 ± 13.1	67.4 ± 13.5^∗^
LDL cholesterol (mg/dL)	99.3 ± 33.2	92.3 ± 26.3	106.1 ± 37.9
Serum triglycerides (mg/dL)	82.2 ± 42.9	92.5 ± 56.3	72.6 ± 22.3

Data are expressed as mean ± SD. ^∗^*p* < 0.05 versus men.

**Table 2:** Energy intake and diet composition of the patients with type 1 diabetes participating in the study.

Nutrient	All (*N* = 60)	Men (*N* = 30)	Women (*N* = 30)	Recommendation^§^
Energy (kcal/day)	1653 ± 367	1842 ± 364	1464 ± 261^∗∗∗^	NA
Alcohol (TE%)	14.9 ± 51	22.4 ± 60.4	7.5 ± 39	NA
Alcohol (g)	2.1 ± 7.3	8.6 ± 1.6	5.6 ± 1	10 M-20 W
Carbohydrate (TE%)	49.3 ± 5.4	51 ± 4.9	47.6 ± 5.5^∗^	45–60
Total soluble sugars (TE%)	14.1 ± 4.2	13.3 ± 4.4	15 ± 3.8	NA
Added sugars (TE%)	2.4 ± 2.8	2.7 ± 3.6	2.1 ± 1.7	<10
Protein (TE%)	17.7 ± 2.4	17.9 ± 2.7	17.5 ± 2.1	10–15
Fat (TE%)	32.8 ± 5.3	30.9 ± 5.1	34.7 ± 4.9^∗∗^	≤35
Saturated fatty acids (TE%)	8.8 ± 2.5	8.4 ± 2.3	9.2 ± 2.7	<10
Monounsaturated fatty acids (TE%)	15.2 ± 3.2	14.5 ± 3.5	15.9 ± 2.7	>10
Polyunsaturated fatty acids (TE%)	3.9 ± 0.8	3.9 ± 0.8	3.9 ± 0.7	<10
Cholesterol (mg)	188 ± 65.6	206.7 ± 62.7	169.3 ± 64.1^∗^	<300
Glycemic Index (%)	55.6 ± 4.6	56.4 ± 4.7	54.8 ± 4.3	45–55
Fiber (g/1000 kcal)	11.8 ± 4.2	11.2 ± 4.2	12.5 ± 4.1	>20
NaCl (g)^+^	4.3 ± 1.5	4.9 ± 1.7	3.8 ± 1.0^∗^	<6

Data are expressed as mean ± SD. ^∗^*p* < 0.05; ^∗∗^*p* < 0.01; ^∗∗∗^*p* < 0.001 versus men. NA = not applicable; TE = total Energy. ^+^Only NaCl present in foods. ^§^Italian Standards of Care ADI-AMD-SID (2013-2014) [14].

**Table 3:** Vitamin and minerals intake expressed per 1000 kcal of total energy intake of the patients with type 1 diabetes participating in the study and patients' adherence to the recommendations for the Italian general population^∗^.

	Men			Women		
	RI/1000 kcal	Intake/1000 kcal	Adherence (%)	RI/1000 kcal	Intake/1000 kcal	Adherence (%)
*Vitamins*						
Vitamin A (*μ*g)	250	440.9 ± 226.1	80	222.2	537.2 ± 250.7	93.3
Vitamin D (*μ*g)	5	1.4 ± 0.9	0	5.5	1.1 ± 0.9	0
Vitamin E (mg)	6.5	5.6 ± 1.5	20	6.7	6.5 ± 1.5	43.3
Thiamine (mg)	0.5	0.5 ± 0.1	66.7	0.5	0.6 ± 0.2	76.7
Riboflavin (mg)	0.6	0.6 ± 0.1	40	0.61	0.7 ± 0.1	80
Vitamin B_6_ (mg)	0.5	0.9 ± 0.2	100	0.61	1 ± 0.3	96.7
Vitamin B_12_ (*μ*g)	1	2.3 ± 1.4	96.7	1.1	2.2 ± 0.9	90
Folate (*μ*g)	160	178.1 ± 76.3	46.7	177.8	196.8 ± 67	52.3
Vitamin C (mg)	37.5	70.7 ± 52	76.7	33.3	86.3 ± 46.8	83.3
Niacin (mg)	7	9.7 ± 2.8	80	7.8	10 ± 2.7	80
*Minerals*						
Calcium (mg)	400	403.6 ± 121.3	50	444.4	493.4 ± 79.0	73.3
Potassium (mg)	1950	1403 ± 352	6.7	2166	1541 ± 316	0
Magnesium (mg)	85	94.5 ± 28.5	63.3	94.4	102.9 ± 24.2	60
Iron (mg)	3.5	5.6 ± 1.9	96.7	5.5	6.1 ± 1.5	56.7
Zinc (mg)	5	5.1 ± 0.8	60	4.4	5.3 ± 1.1	76.7
Copper (mg)	0.3	0.4 ± 0.1	66.7	0.4	0.4 ± 0.1	63.3
Selenium (*μ*g)	22.5	18.8 ± 6.5	23.3	25	20.2 ± 8.2	23.3

Data are expressed as mean ± SD. RI: recommended intake. ^∗^Società Italiana di Nutrizione Umana. Livelli di Assunzione di Riferimento di Nutrienti ed Energia per la popolazione italiana (LARN–IV review 2014) [16].

## References

[1] Bailey R. L., West K. P., Black R. E. (2015). The epidemiology of global micronutrient deficiencies. *Annals of Nutrition & Metabolism*.

[2] Rautiainen S., Manson J. E., Lichtenstein A. H., Sesso H. D. (2016). Dietary supplements and disease prevention -- a global overview. *Nature Reviews Endocrinology*.

[3] Lin C. C., Huang Y. L. (2015). Chromium, zinc and magnesium status in type 1 diabetes. *Current Opinion in Clinical Nutrition and Metabolic Care*.

[4] Sette S., Le Donne C., Piccinelli R. (2011). The third Italian National Food Consumption Survey, INRAN-SCAI 2005-06 e Part 1: nutrient intakes in Italy. *Nutrition, Metabolism and Cardiovascular Diseases*.

[5] Randecker G. A., Smiciklas-Wright H., McKenzie J. M. (1996). The dietary intake of children with IDDM. *Diabetes Care*.

[6] Ahola A. J., Mikkilä V., Mäkimattila S. (2012). Energy and nutrient intakes and adherence to dietary guidelines among Finnish adults with type 1 diabetes. *Annals of Medicine*.

[7] Parillo M., Annuzzi G., Rivellese A. A. (2011). Effects of meals with different glycaemic index on postprandial blood glucose response in patients with type 1 diabetes treated with continuous subcutaneous insulin infusion. *Diabetic Medicine*.

[8] Bozzetto L., Giorgini M., Alderisio A. (2015). Glycaemic load versus carbohydrate counting for insulin bolus calculation in patients with type 1 diabetes on insulin pump. *Acta Diabetologica*.

[9] Bozzetto L., Alderisio A., Giorgini M. (2016). Extra-virgin olive oil reduces glycemic response to a high-glycemic index meal in patients with type 1 diabetes: a randomized controlled trial. *Diabetes Care*.

[10] Toeller M., Klischan A., Heitkamp G. (1996). Nutritional intake of 2868 IDDM patients from 30 centres in Europe. *Diabetologia*.

[11] Snell-Bergeon J. K., Chartier-Logan C., Maahs D. M. (2009). Adults with type 1 diabetes eat a high-fat atherogenic diet which is associated with coronary artery calcium. *Diabetologia*.

[12] Diabetes and Nutrition Study Group of the Spanish Diabetes Association (GSEDNu) (2006). Diabetes Nutrition and Complications Trial: adherence to the ADA nutritional recommendations, targets of metabolic control, and onset of diabetes complications. A 7-year, prospective, population-based, observational multicenter study. *Journal of Diabetes and its Complications*.

[13] Carnovale E., Marletta L. (2000). *Tabelle di Composizione degli Alimenti - Aggiornamento 2000 INRAN*.

[14] Associazione Medici Diabetologi (AMD) (2014). *Standard italiani per la cura del diabete mellito 2013-2014*.

[15] Mann J. I., De Leeuw I., Hermansen K. (2004). Evidence-based nutritional approaches to the treatment and prevention of diabetes mellitus. *Nutrition, Metabolism, and Cardiovascular Diseases*.

[16] Società Italiana di Nutrizione Umana (2014). *Livelli di Assunzione di Riferimento di Nutrienti ed Energia per la popolazione italiana (LARN)*.

[17] Rivellese A. A., Boemi M., Cavalot F. (2008). Dietary habits in type II diabetes mellitus: how is adherence to dietary recommendations?. *European Journal of Clinical Nutrition*.

[18] Vitale M., Masulli M., Cocozza S. (2016). Sex differences in food choices, adherence to dietary recommendations and plasma lipid profile in type 2 diabetes – the TOSCA.IT study. *Nutrition, Metabolism, and Cardiovascular Diseases*.

[19] Pietinen P., Paturi M., Reinivuo H., Tapanainen H., Valsta M. L. (2010). FINDIET 2007 Survey: energy and nutrient intakes. *Public Health Nutrition*.

[20] US Department of Agriculture (2010). *Dietary Guidelines for Americans, 2010*.

[21] Quann E. E., Fulgoni V. L., Auestad N. (2015). Consuming the daily recommended amounts of dairy products would reduce the prevalence of inadequate micronutrient intakes in the United States: diet modeling study based on NHANES2007-2010. *Nutrition Journal*.

